# Oxygen vacancies controlled multiple magnetic phases in epitaxial single crystal Co_0.5_(Mg_0.55_Zn_0.45_)_0.5_O_1-*v*_ thin films

**DOI:** 10.1038/srep24188

**Published:** 2016-04-11

**Authors:** Dapeng Zhu, Qiang Cao, Ruimin Qiao, Shimeng Zhu, Wanli Yang, Weixing Xia, Yufeng Tian, Guolei Liu, Shishen Yan

**Affiliations:** 1 School of Physics, State Key Laboratory of Crystal Materials, Shandong University, Jinan, 250100, P. R. China; 2 Advanced Light Source, Lawrence Berkeley National Laboratory, Berkeley, CA 94720, USA; 3 Ningbo Institute of Materials Technology and Engineering, Chinese Academy of Sciences, Ningbo, 315201, P. R. China

## Abstract

High quality single-crystal *fcc*-Co_*x*_(Mg_*y*_Zn_1-*y*_)_1-*x*_O_1-*v*_ epitaxial thin films with high Co concentration up to x = 0.5 have been fabricated by molecular beam epitaxy. Systematic magnetic property characterization and soft X-ray absorption spectroscopy analysis indicate that the coexistence of ferromagnetic regions, superparamagnetic clusters, and non-magnetic boundaries in the as-prepared Co_*x*_(Mg_*y*_Zn_1-*y*_)_1-*x*_O_1-*v*_ films is a consequence of the intrinsic inhomogeneous distribution of oxygen vacancies. Furthermore, the relative strength of multiple phases could be modulated by controlling the oxygen partial pressure during sample preparation. Armed with both controllable magnetic properties and tunable band-gap, Co_*x*_(Mg_*y*_Zn_1-*y*_)_1-*x*_O_1-*v*_ films may have promising applications in future spintronics.

Magnetic oxides comprise a wide class of materials exhibiting rich crystal structures and physical properties that make them ideal candidates for both theoretical and experimental studies^1^. The interest in magnetic oxides has exponentially grown, stimulated by the discovery of high temperature superconductivity in cuprates^2^, colossal magnetoresistance in mixed valence manganese oxides^3^ and above room temperature ferromagnetism in wide-band-gap oxide ferromagnetic semiconductors^4^,^5^,^6^. In particular, the experimental observation of phase separation and multiple phase coexistence in oxides is of great interest owing to the fact that phase coexistence can result in novel electronic and/or magnetic properties.

The coexistence of distinct metallic and insulating electronics phase in pervoskite magnetic oxides presents researcher a tool to tune the electronic properties of materials where metal-insulator transition accompanied with colossal magnetoresistance effect could be achieved^3^,^7^,^8^. The coexistence of superconductivity and ferromagnetism at the interface between two oxide insulators provides a fascinating system for the study of the interplay between superconductivity and magnetism^9^,^10^,^11^, because ferromagnetism is usually considered to be incompatible with conventional superconductivity, as it destroys the singlet correlations responsible for the pairing interaction. Also, the coexistence of competing magnetic phases in the complex oxide heterojunctions offers excellent opportunities to exploit emerging magnetic phenomena such as spin glass and exchange bias effect^12^,^13^. Generally, in the case of strongly correlated oxide systems, the orbital selective occupancy, Coulomb interaction, Hund coupling and Jahn-Teller distortions have a significant role in determining the nature of the electronic and magnetic states. However, the intricate relationship between spin, charge and orbit degree of freedom in the strongly correlated oxide system leads to rich phase diagram and makes the understanding of multiple phase coexistence rather complicated. Alternatively, an in-depth understanding of the microscopic origin of multiple phase coexistence could be achieved in oxide systems with less complexity.

Here we pay special attention to the ferromagnetic oxide semiconductors, such as diluted magnetic oxides^14^,^15^,^16^ (TM-doped ZnO and TM-doped TiO_2_ etc., where TM = Mn, Fe, Co transitional metal), diluted magnetic dielectrics^17^,^18^,^19^ (TM-doped CeO_2_ and Sm_2_O_3_ etc.), condensed oxide ferromagnetic semiconductors^5^ and even in undoped wide band gap oxides^20^,^21^. Different from the strongly correlated oxide systems, exchange interactions mainly in the form of *s,p-d* hybridizing play the definitive role in determining the final magnetic phases of oxide ferromagnetic semiconductors, which present a new platform to study multiple phase coexistence. Unfortunately, up to date, high quality single crystal magnetic oxides with high TM concentrations have not been prepared. In order to achieve this goal, we choose to investigate quaternary *fcc*-Co_*x*_(Mg_*y*_Zn_1-*y*_)_1-*x*_O_1-*v*_ (CoMgZnO) epitaxial thin films. Though secondary phases were usually detected in the Co_x_Zn_1-x_O films when the Co concentration is above 25%^22^,^23^, ternary Mg_x_Zn_1-x_O shows structural evolution from ZnO-based hexagonal structure to MgO-based face-centered-cubic structure with increasing Mg concentration^24^. In addition, MgO-based *fcc* structure matches well with CoO and Co_x_Mg_1-x_O is available over the entire composition range^25^. Hence, quaternary *fcc*-Co_*x*_(Mg_*y*_Zn_1-*y*_)_1-*x*_O_1-*v*_ provides an alternative way to break the low solubility limitation of transitional metal. Moreover, band gap engineering could be expected in CoMgZnO by tuning the composition ratio of Mg/Zn. Above all, magnetic property modulation could be achieved by tuning the Co and oxygen vacancy concentration in the CoMgZnO films, which makes CoMgZnO a promising candidate for future optical and spintronics applications.

In this report, we demonstrated that single crystal *fcc*-Co_*x*_(Mg_*y*_Zn_1-*y*_)_1-*x*_O_1-*v*_ with Co concentration up to x = 0.5 has been fabricated for the first time. The systematic magnetic property and X-ray absorption spectroscopy (XAS) measurements indicated that intrinsic inhomogeneous distribution of oxygen vacancies leads to the coexistence of ferromagnetic, superparamagnetic and non-magnetic phases in the as-prepared CoMgZnO epitaxial films. In addition, the relative strength of multiple phases could be modulated by controlling the oxygen partial pressure during sample preparation.

## Results

### Microstructure of single-crystal thin films

**Figure 1:**
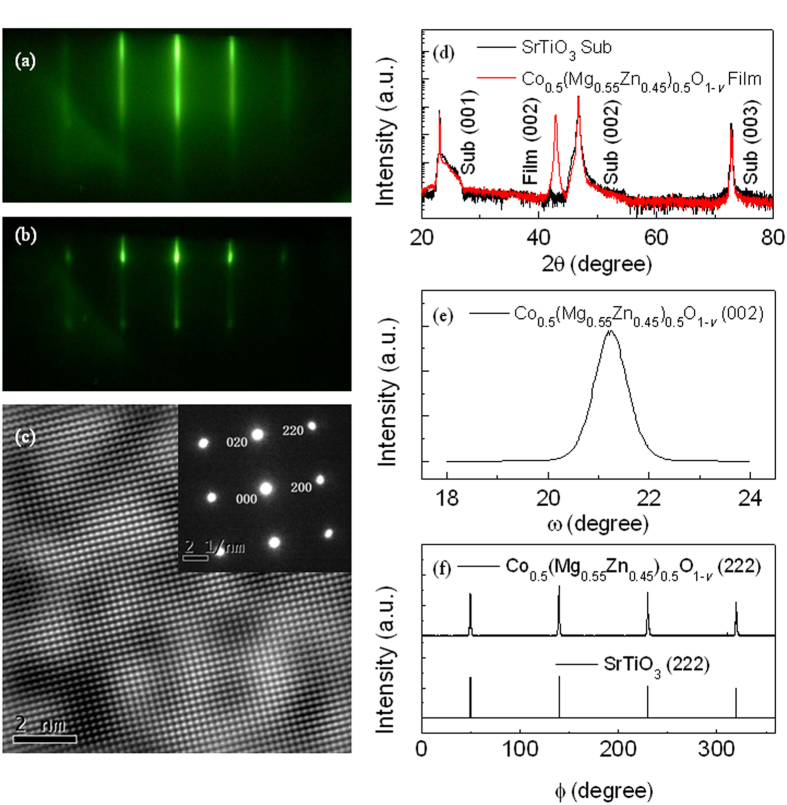
Microstructure of epitaxial single-crystal Co_0.5_(Mg_0.55_Zn_0.45_)_0.5_O_1-*v*_ thin films. (**a**) and (**b**) RHEED patterns taken along the [110] azimuth for the MgO buffer layer and Co_0.5_(Mg_0.55_Zn_0.45_)_0.5_O_1-*v*_ films. (**c**) High resolution TEM of the Co_0.5_(Mg_0.55_Zn_0.45_)_0.5_O_1-*v*_ films, and the inset of (**c**) shows the SAED results. (**d**) XRD θ-2θ spectra of the SrTiO_3_ substrate and Co_0.5_(Mg_0.55_Zn_0.45_)_0.5_O_1-*v*_ films. (**e**) XRD rocking curve of the Co_0.5_(Mg_0.55_Zn_0.45_)_0.5_O_1-*v*_ (002) peak. (f) XRD ϕ scans of the SrTiO_3_ (222) and Co_0.5_(Mg_0.55_Zn_0.45_)_0.5_O_1-*v*_ (222) planes.

Typical RHEED patterns of MgO buffer layer deposited on SrTiO_3_ (001) substrate were shown in Fig. 1(a), demonstrating the well flatness of the growth surface, which provides fine template for later epitaxial growth. In Fig. 1(b), streaky RHEED patterns were observed for the Co_0.5_(Mg_0.55_Zn_0.45_)_0.5_O_1-*v*_ films prepared under oxygen partial pressure of 6 × 10^−7^ mbar, indicating a two-dimensional plus three-dimensional growth mode. No secondary phase related spots appeared in RHEED patterns for all the films, excluding the presence of impurity precipitations. High resolution TEM image and selected area electron diffraction (SAED) of the Co_0.5_(Mg_0.55_Zn_0.45_)_0.5_O_1-*v*_ films were further shown in Fig. 1(c), which indicated that high quality single crystalline CoMgZnO films without any sign of secondary phases within the detection limit have been synthesized. The dark/bright contrast arising from local stress or inhomogeneous composition distribution can be observed, which suggest that elements distribution may be not uniform on nanometer scale.

To further confirm the high quality single-crystal structure of the studied Co_0.5_(Mg_0.55_Zn_0.45_)_0.5_O_1-*v*_ films, X-ray diffraction (XRD) measurements were performed. In Fig. 1(d), only the (002) peak of Co_0.5_(Mg_0.55_Zn_0.45_)_0.5_O_1-*v*_ films was found besides the substrate peaks, excluding any secondary phase. Figure 1(e) shows the XRD omega rocking curve of the Co_0.5_(Mg_0.55_Zn_0.45_)_0.5_O_1-*v*_ (002) peak, and a full width at half maximum of 0.82° was obtained from the Gaussian fitting. By using the Scherrer equation 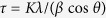, the estimated crystal coherence length 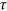 is about 35 nm with 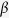 = Δ(2θ) = 0.245° = 0.0043 rad and θ  = 21.5°, certifying the high crystal quality.

The XRD ϕ scans of the SrTiO_3_ (222) and Co_0.5_(Mg_0.55_Zn_0.45_)_0.5_O_1-*v*_ (222) planes were shown in Fig. 1(f). Four sharp peaks with 90° apart indicate in-plane four-fold symmetry for both the Co_0.5_(Mg_0.55_Zn_0.45_)_0.5_O_1-*v*_ films and SrTiO_3_ substrate. From the XRD results and RHEED patterns, the cubic-on-cubic epitaxial relationship of Co_0.5_(Mg_0.55_Zn_0.45_)_0.5_O_1-*v*_ (001)[100] // MgO (001)[100] // SrTiO_3_ (001)[100] is confirmed. So it is clear that we have prepared the high quality epitaxial single-crystal Co_0.5_(Mg_0.55_Zn_0.45_)_0.5_O_1-*v*_ thin films with high Co concentration.

### Multiple magnetic phases

**Figure 2:**
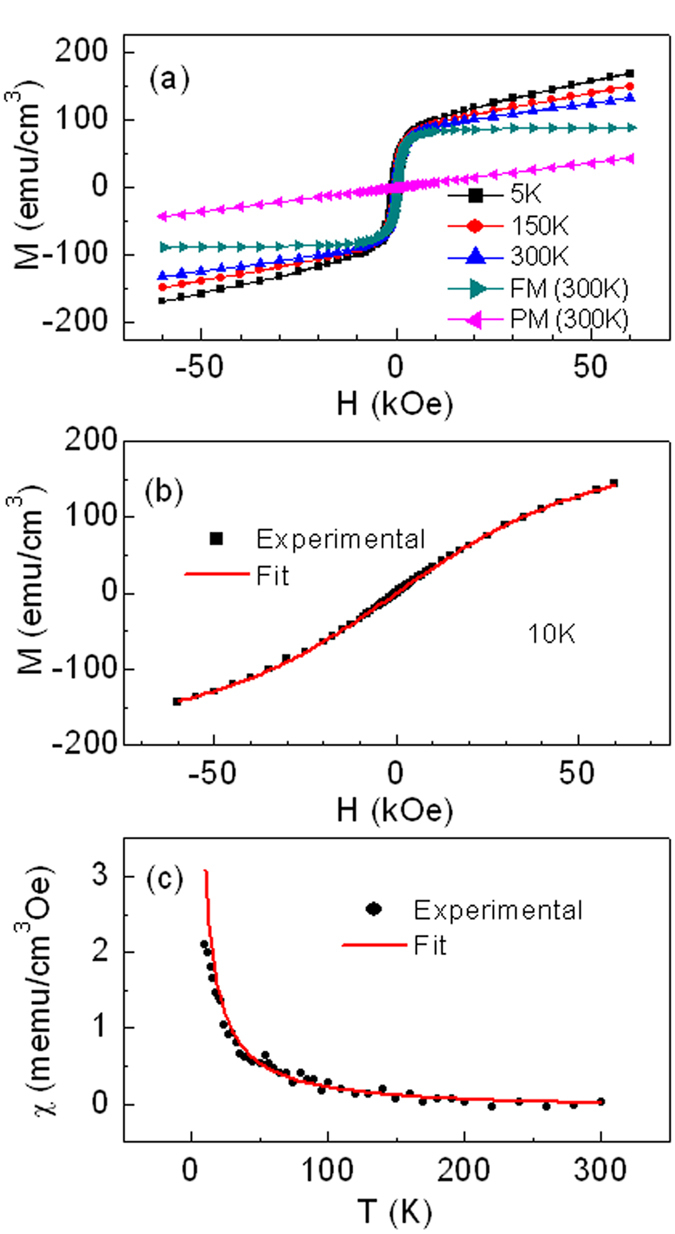
Magnetic properties of Co_0.5_(Mg_0.55_Zn_0.45_)_0.5_O_1-*v*_ films. (**a**) *M-H* curves of the as-prepared Co_0.5_(Mg_0.55_Zn_0.45_)_0.5_O_1-*v*_ films measured at 5, 150 and 300K, together with the separated ferromagnetic (FM) and superparamagnetic (PM) contributions at 300K. (**b**) *M-H* curve of the annealed Co_0.5_(Mg_0.55_Zn_0.45_)_0.5_O_1-*v*_ films measured at 10 K and the fitting *M-H* curve by Langevin function. (**c**) Temperature dependence of magnetic susceptibility of the annealed Co_0.5_(Mg_0.55_Zn_0.45_)_0.5_O_1-*v*_ films and the fitting curve by Curie law.

Figure 2(a) shows in-plane magnetic field dependence of magnetization (*M-H* curves) for the Co_0.5_(Mg_0.55_Zn_0.45_)_0.5_O_1-*v*_ films measured by SQUID at 5, 150 and 300 K. As can be seen, ferromagnetism is clearly observed from the hysteresis loops at low magnetic field, which only shows slight change within the studied temperature range, while paramagnetic and/or superparamagnetic response are observed at high magnetic field and decreases obviously with increasing temperature. Therefore, the room temperature ferromagnetic and paramagnetic/superparamagnetic contributions were separated and displayed in Fig. 2(a). The saturation magnetization (*M*_*s*_) of the ferromagnetic component is 86.3 emu/cm^3^, which is much higher as compared with generally low values in diluted magnetic semiconductors^14^,^15^,^26^. Such a large saturation magnetization is highly unlikely to be from small clusters of metallic constitutes which are beyond the detection limit of commercial XRD, RHEED and HRTEM. In addition, almost no magnetic anisotropy was observed in spite of the single-crystal structure.

We further investigated the magnetic properties of Co_0.5_(Mg_0.55_Zn_0.45_)_0.5_O_1-*v*_ films which were annealed at 550 °C for 2 h under O_2_ atmosphere in a tube furnace. Figure 2(b) shows the *M-H* curve of the annealed films measured at 10 K. It is clear that the paramagnetic/superparamagnetic *M-H* curve was observed instead of ferromagnetic hysteresis loops. Therefore, the experimental *M-H* curve was fitted by Langevin function


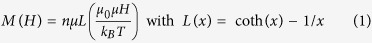


where *n* is the density of magnetic clusters, *μ* is the average magnetic moments per cluster, *μ*_*0*_ is the permeability of vacuum, *k*_*B*_ is the Boltzmann constant, and *T* is the absolute temperature. A very good fitting to the experimental *M-H* curve gives *n* = 4.43 × 10^15 ^cm^−3^, *μ* = 4.99 × 10^−14 ^emu = 5.38 × 10^6 ^*μ*_*B*_, and the saturation magnetization *M*_*s*_ = *nμ* = 221.1 emu/cm^3^. This means that the annealed single crystal films are composed of superparamagnetic clusters with the magnetic moments *μ* = 5.38 × 10^6 ^*μ*_*B*_ per cluster and the density of superparamagnetic clusters *n* = 4.43 × 10^15 ^cm^−3^. On the other hand, if we assume these superparamagnetic clusters coalesce together, they will become ferromagnetic. Therefore, these superparamagnetic clusters should be well separated by non-magnetic boundaries. Assuming all Co atoms disperse uniformly in the superparamagnetic clusters and have the same magnetic moments, the average magnetic moments per Co atom were derived to be 0.90 *μ*_*B*_. In this sense, according to Eq. (1), the total contribution of isolated paramagnetic Co atomic moments (if exist) is negligible for our magnetic measurements as compared with the contribution of superparamagnetic clusters.

Figure 2(c) shows the temperature dependence of magnetic susceptibility of the annealed films as well as the fitting curve by the Curie law


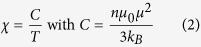


where *C* is the Curie constant. It can be seen that the fitting curve generally coincides with the experimental data, and the Curie constant *C* = 3.16 × 10^−2^ (emu·K)/(cm^3^·Oe) was obtained. On the other hand, using the parameters *n* and *μ* of Langevin fitting of the *M-H* curve in Fig. 2(b), we can directly obtain the Curie constant *C* = 3.35 × 10^−2^ (emu·K)/(cm^3^·Oe), which is well consistent with the *χ-T* curve fitting. This further indicates that the experimentally measured magnetic susceptibility is attributed to the superparamagnetic clusters and the contribution of isolated paramagnetic Co atoms (if exist) is negligible.

**Figure 3:**
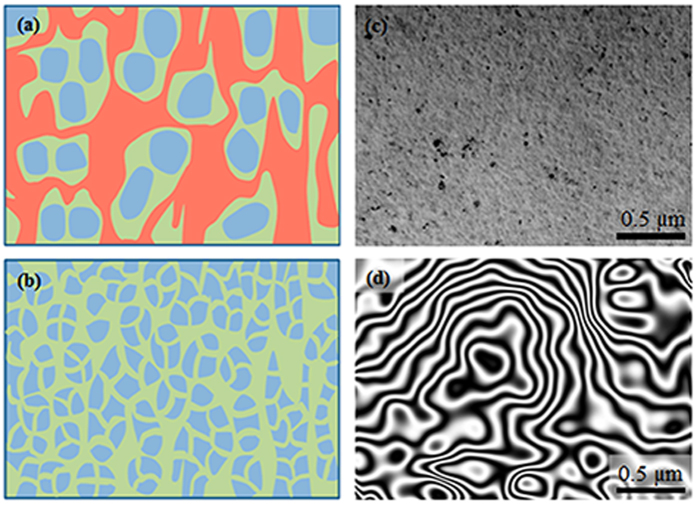
Multiple magnetic phases coexistence. (**a**) and (**b**) Schematic diagrams of coexistence of three magnetic phases in the as-prepared and oxygen annealed Co_0.5_(Mg_0.55_Zn_0.45_)_0.5_O_1-*v*_ films. The red, blue, and green regions represent for ferromagnetic regions, superparamagnetic clusters, and non-magnetic boundaries, respectively. (**c**) Bright field image and (**d**) reconstructed phase image of electron holography of the as-prepared Co_0.5_(Mg_0.55_Zn_0.45_)_0.5_O_1-*v*_ films.

Now let us turn back to Fig. 2(a) to further reveal the magnetic properties of the as-prepared Co_0.5_(Mg_0.55_Zn_0.45_)_0.5_O_1-*v*_ films. We assume all Co atoms are in the ferromagnetic and superparamagnetic clusters in the as-prepared Co_0.5_(Mg_0.55_Zn_0.45_)_0.5_O_1-*v*_ films and have the same magnetic moments per Co atom as that in the oxygen annealed films, i.e., 0.90 *μ*_*B*_ per Co atom corresponding to the total saturation magnetization of *M*_*s*_ = 221.1 emu/cm^3^. We can derive the saturation magnetization of the superparamagnetic component *nμ* = 134.8 emu/cm^3^ by deducting the ferromagnetic component 86.3 emu/cm^3^ from the total saturation magnetization of *M*_*s*_ = 221.1 emu/cm^3^. Furthermore, from the experimental magnetic susceptibility *χ* of the superparamagnetic component in Fig. 2(a), the density of superparamagnetic clusters *n* = 2.44 × 10^14 ^cm^−3^ and average magnetic moments per cluster *μ* = 5.72 × 10^7 ^*μ*_*B*_ can be calculated by Eq. (2). This means that the as-prepared Co_0.5_(Mg_0.55_Zn_0.45_)_0.5_O_1-*v*_ films are composed of ferromagnetic regions, superparamagnetic clusters, and non-magnetic boundaries. The schematic diagrams of the coexistence of three different magnetic phases in the as-prepared films were shown in Fig. 3(a). Comparing the density of superparamagnetic clusters *n* and average magnetic moments per cluster *μ* between the as-prepared Co_0.5_(Mg_0.55_Zn_0.45_)_0.5_O_1-*v*_ films (*n* = 2.44 × 10^14 ^cm^−3^, *μ* = 5.72 × 10^7 ^*μ*_*B*_) and the oxygen annealed films (*n* = 4.43 × 10^15 ^cm^−3^, *μ* = 5.38 × 10^6 ^*μ*_*B*_), we found that both ferromagnetic regions and superparamagetic clusters in the as-prepared films involve into much smaller superparamagetic clusters with more density of superparamagnetic clusters and less magnetic moments per cluster after oxygen annealing, as shown in Fig. 3(b).

Electron holography experiments were performed and Fig. 3(c) and Fig. 3(d) respectively show the bright field image and reconstructed phase image of the as-prepared Co_0.5_(Mg_0.55_Zn_0.45_)_0.5_O_1-*v*_ films in the remanent magnetization states. The bright filed image (Fig. 3(c)) shows uniform morphology, which is consistent with the high quality single crystal structure in Fig. 1 (c). However, complex magnetic patterns are revealed by the reconstructed phase image in Fig. 3(d), suggesting the coexistence of multiple magnetic phases despite the structural uniformity. In the reconstructed phase image the black (or white) lines are the lines of magnetic flux, i.e., the tangent line and the density of which represent the direction and strength of the magnetic flux, respectively. Evident lines are observed and the lines are irregularly distributed, which mean the ferromagnetic characteristic and inhomogeneous magnetization. If the as-prepared Co_0.5_(Mg_0.55_Zn_0.45_)_0.5_O_1-*v*_ films are nonmagnetic or superparamagnetic, no lines will exist. If the films are uniformly ferromagnetic, the space between lines should be same and the lines should be more regularly distributed, e.g. almost parallel in some directions due to small coercivity. It is shown that the lines go through the ferromagnetic regions and are curved by the nonmagnetic or superparamagnetic regions, which revealed that the as-prepared Co_0.5_(Mg_0.55_Zn_0.45_)_0.5_O_1-*v*_ films are composed of ferromagnetic regions, superparamagnetic clusters, and non-magnetic boundaries. This scenario revealed by the reconstructed phase image is in well agreement with the magnetic measurements by SQUID, as schematically shown in Fig. 3(a).

### Controllable ferromagnetism

**Figure 4:**
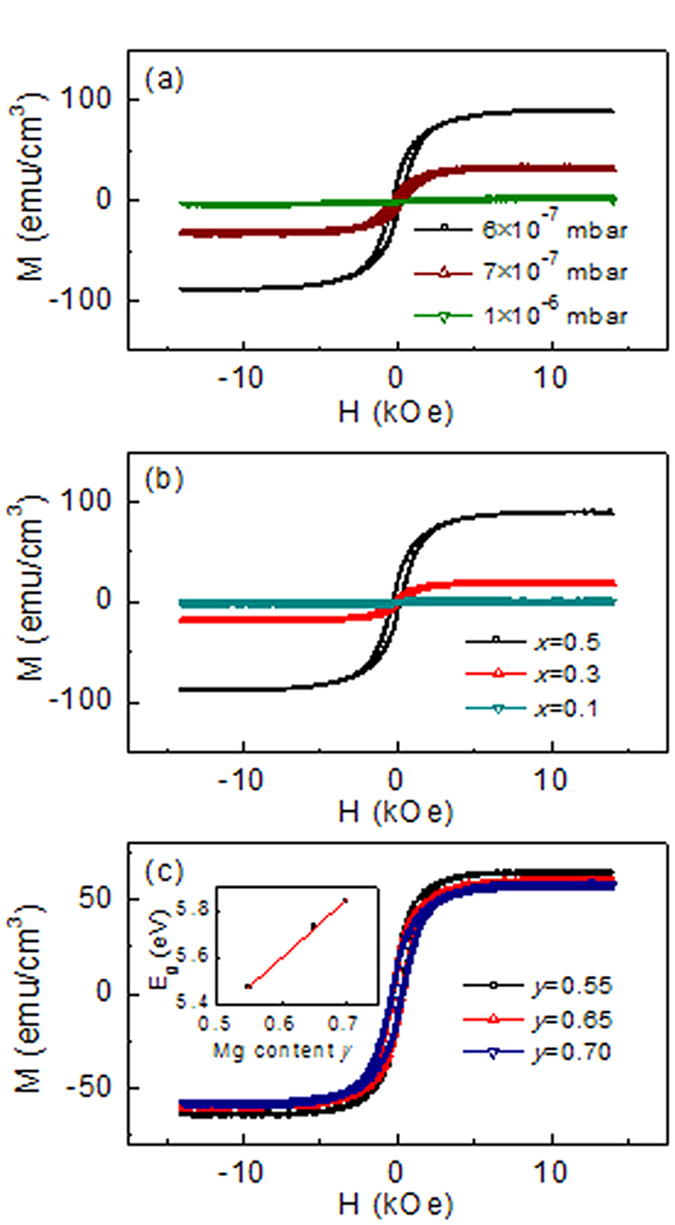
Controllable magnetic properties. (**a**) *M-H* curves of Co_0.5_(Mg_0.55_Zn_0.45_)_0.5_O_1-*v*_ films fabricated on SrTiO_3_ substrate under different oxygen partial pressure. (**b**) *M-H* curves of Co_*x*_(Mg_0.55_Zn_0.45_)_1-*x*_O_1-*v*_ films with various Co concentration grown on SrTiO_3_ substrate. (**c**) *M-H* curves of Co_0.5_(Mg_*y*_Zn_1-*y*_)_0.5_O_1-*v*_ films with different Mg content grown on sapphire substrate. The inset of (**c**) shows the increased band gap with increasing Mg concentration and the linear fitting.

Furthermore, the room temperature ferromagnetism was modulated by controlling the preparation oxygen partial pressure and chemical composition. Since the superparamagnetic signals were easily deducted from *M-H* curves, we just show the ferromagnetic *M-H* loops in Fig. 4. Figure 4(a) shows room temperature ferromagnetic *M-H* loops of Co_0.5_(Mg_0.55_Zn_0.45_)_0.5_O_1-*v*_ films fabricated under different oxygen partial pressure (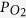). The ferromagnetic saturation magnetization *M*_*s*_ decreases from 86.3 to 32.7 emu/cm^3^ with increasing 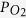 from 6 × 10^−7^ to 7 × 10^−7^ mbar, and then decreased to 2.7 emu/cm^3^ with increasing 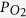 to 1 × 10^−6^ mbar. It is believed that changing the oxygen partial pressure during preparation or post annealing in oxygen could change the density of oxygen vacancies in the films, and thus change the magnetism.

Figure 4(b) shows room temperature ferromagnetic *M-H* loops of Co_*x*_(Mg_0.55_Zn_0.45_)_1-*x*_O_1-*v*_ films with various Co concentration prepared under oxygen partial pressure of 6 × 10^−7^ mbar. It is found that the ferromagnetic *M*_*s*_ decreases from 86.3 to 18.2 emu/cm^3^ with decreasing *x* from 0.5 to 0.3, and then decreases to almost 0 emu/cm^3^ with *x* decreased to 0.1. This is consistent with the scenario that Co provides the local magnetic moments and oxygen vacancies mediate the exchange coupling between local magnetic moments. As a result of the intrinsic inhomogeneous distribution of oxygen vacancies, regions with high oxygen vacancies concentration become ferromagnetic and regions with low oxygen vacancies concentration show superparamagnetic or non-magnetic behavior. However, when Co concentration is below the threshold value (x < 0.1), ferromagnetic order could not be established in our preparation conditions. Figure 4(c) shows room temperature ferromagnetic *M-H* curves of various Co_0.5_(Mg_*y*_Zn_1-*y*_)_0.5_O_1-*v*_ films, which were fabricated on Al_2_O_3_ (0001) substrates under oxygen partial pressure of 6 × 10^−7^ mbar. For these epitaxial films, the [111] direction of CoMgZnO is parallel to the c-axis of sapphire. All these films show room temperature ferromagnetism, and the ferromagnetic *M*_*s*_ (≈64.6 emu/cm^3^) is almost unchanged with increasing the Mg concentration from 0.55 to 0.70. However, it is about 25% smaller than that (86.3 emu/cm^3^) of films grown on SrTiO_3_. The decrease of *M*_*s*_ suggests that the actual oxygen vacancies density should be different for films grown on different substrates. The optical transmittance spectra were measured to obtain the band gap of Co_0.5_(Mg_*y*_Zn_1-*y*_)_0.5_O_1-*v*_ films grown on Al_2_O_3_ substrates. Here Al_2_O_3_ (0001) substrate with larger band gap is beneficial for measuring the band gap of the Co_0.5_(Mg_*y*_Zn_1-*y*_)_0.5_O_1-*v*_ films. The dependence of band gap *E*_*g*_ on the Mg content *y* was shown in the inset of Fig. 4(c), and a linear fit yields *E*_*g*_ = (2.49*y *+ 4.11) eV, which is a little smaller than that of pure Mg_*y*_Zn_1-*y*_O films^27^. This may be induced by the presence of Co and/or the inhomogeneous Mg distribution^28^, because the band gap of CoO is smaller than that of MgO.

All these results indicate that the final magnetic phases, i.e., the relative strength of the ferromagnetism, superparamagnetism, and nonmagnetic phases, could be modulated on large scale simply through controlling the oxygen partial pressure during sample growth and changing the chemical composition. However, high Co concentration and sufficient oxygen vacancies are crucial to realize strong ferromagnetism.

### Oxygen vacancies and ferromagnetism revealed by X-ray absorption spectrum

**Figure 5:**
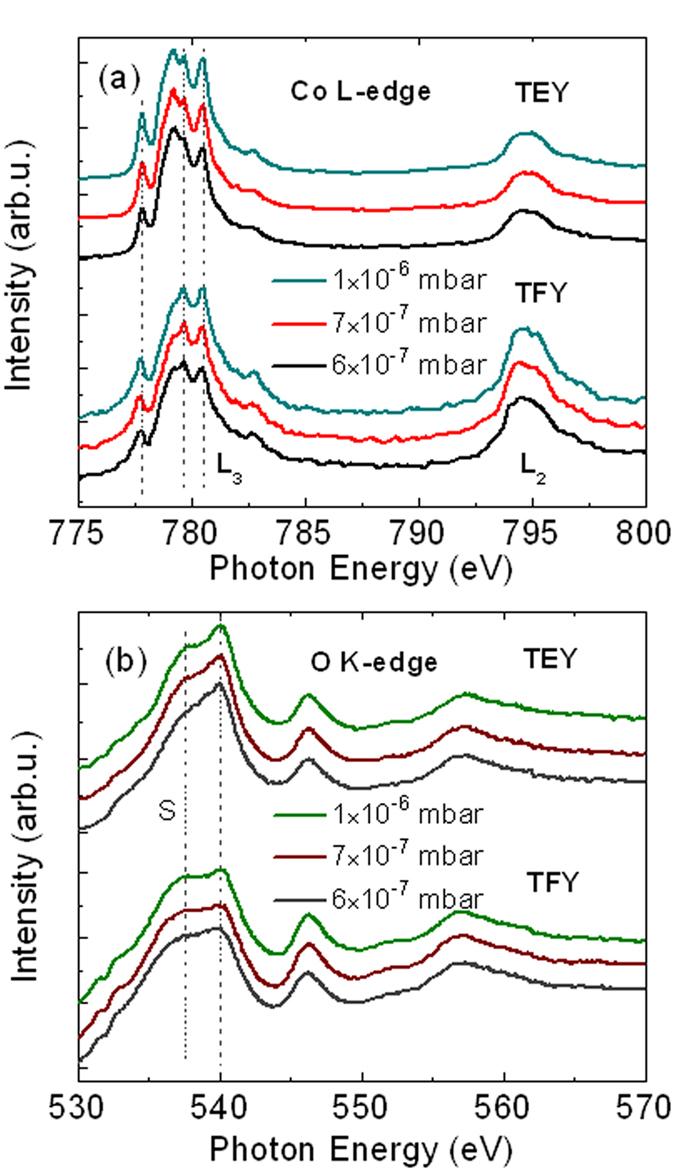
X-ray absorption spectrum. Co *L*-edge XAS (**a**) and O *K*-edge XAS (**b**) of the Co_0.5_(Mg_0.55_Zn_0.45_)_0.5_O_1-*v*_ films fabricated under different oxygen partial pressure.

Now we further reveal the evolution of ferromagnetism by X-ray absorption spectrum. It is well known that XAS is very sensitive to the formation of native defects and site symmetry of specific atoms in materials, and hence has great advantages in probing the local electronic structure^29^,^30^. Figure 5(a) shows the Co *L*-edge XAS of the Co_0.5_(Mg_0.55_Zn_0.45_)_0.5_O_1-*v*_ films fabricated under different oxygen partial pressure, where both surface-sensitive total electron yield (TEY) and bulk-sensitive total fluorescence yield (TFY) modes give almost the same results. We can see that the Co *L*_*3*_ and *L*_*2*_ absorption peaks are located at 780 eV and 795 eV, respectively, which are separated by the 2*p* core-hole spin-orbit interaction. The general line shape of all the acquired spectra shows characteristic features similar to those in CoO^31^, and is in good agreement with the line shape of Co^2+^ ion in octahedral cluster calculated by charge-transfer multiplet model^32^. This indicates that the Co dopants reside cationic sites and octahedrally coordinates with ligand O atoms.

However, as compared with the main peak at 779.7 eV, systematic degradation of the features at 777.8 eV and 780.5 eV can be observed with decreasing the oxygen partial pressure (increasing the oxygen vacancies). Here, the three measured Co_0.5_(Mg_0.55_Zn_0.45_)_0.5_O_1-*v*_ films have the same crystal structure, film thickness, and (Co,Mg,Zn) composition. The only difference between them is the oxygen partial pressure during preparation, which leads to the increased oxygen vacancies with decreasing oxygen partial pressure. In this sense, the increased oxygen vacancies would affect the local surrounding of Co ions and hence the electronic band structure, leading to the systemic evolution of XAS features at 777.8 eV and 780.5 eV.

Figure 5(b) further shows the O *K*-edge XAS of the Co_0.5_(Mg_0.55_Zn_0.45_)_0.5_O_1-*v*_ films, where the general line shape of the spectra are similar for all the three samples. The O *K*-edge XAS spectra involves the O 1*s*→2*p* transition, and the features near the edge arise from the hybridization between cationic states and oxygen 2*p* states (*p-d* hybridization), which reduces the number of filled O 2*p* orbitals and enables the dipole transition^33^. It should be noted that a shoulder at 537.6 eV (as denoted with S) was observed for all the films, which degrades with decreasing the oxygen partial pressure (increasing the oxygen vacancies). Previous first-principle calculations have revealed that the presence of oxygen vacancies will result in the reduction and broadening of similar shoulder in O *K*-edge XAS of Co-doped ZnO system^30^. As mentioned above, the only difference between the three samples is the density of oxygen vacancies. Thus, the degradation of the shoulder feature at 537.6 eV with decreasing oxygen partial pressure should be induced by the increased oxygen vacancies.

Analysis of both the Co *L*-edge and O *K*-edge XAS reveals the presence of oxygen vacancies in the Co_0.5_(Mg_0.55_Zn_0.45_)_0.5_O_1-*v*_ films, which increases with decreasing the oxygen partial pressure. Considering magnetic properties measurements have revealed the enhancement of ferromagnetism with decreasing oxygen partial pressure, the XAS results further confirmed the close correlation between the oxygen vacancies and ferromagnetism in the films. These results strongly support the ferromagnetic scenario that oxygen-vacancies-mediated exchange coupling between Co spins is responsible for the ferromagnetism in CoMgZnO thin films, as proposed in the framework such as bound magnetic polaron model and charge transfer model^16^,^34^. However, since oxygen vacancies are very local, the ferromagnetic ordering mediated through oxygen vacancies are also local and can be regarded as superparamagnetic clusters, as shown by the schematic diagrams in Fig. 3(b). Only when these clusters coalesce together, the intrinsic and long-range ferromagnetism can be established, as shown in Fig. 3(a). In this sense, the non-magnetic boundaries are the areas without oxygen vacancies. Therefore, the coexistence of ferromagnetic regions, superparamagnetic clusters, and non-magnetic boundaries in the as-prepared CoMgZnO films indicates that there exists intrinsic inhomogeneous distribution of oxygen vacancies.

## Discussion

Epitaxial single-crystal *fcc*-Co_*x*_(Mg_*y*_Zn_1-*y*_)_1-*x*_O_1-*v*_ thin films with high Co concentration up to *x* = 0.5 have been successfully fabricated by molecular beam epitaxy. Three different magnetic phases of ferromagnetic regions, superparamagnetic clusters, and non-magnetic boundaries were found to coexist in the single-crystal Co_0.5_(Mg_0.55_Zn_0.45_)_0.5_O_1-*v*_ thin films, and they can be modulated on large scale by controlling oxygen vacancies concentration. All the experimental results indicate that intrinsic inhomogeneous distribution of oxygen vacancies plays the definitive role in determining the final magnetic phases in the Co_*x*_(Mg_*y*_Zn_1-*y*_)_1-*x*_O_1-*v*_ films. At last, it should be pointed out that armed with tunable ferromagnetism and band-gap, Co_*x*_(Mg_*y*_Zn_1-*y*_)_1-*x*_O_1-*v*_ films hold promise for future spintronic applications, such as spin-LED and spin-FET^35^,^36^. On the other hand, the superparamagnetic Co_*x*_(Mg_*y*_Zn_1-*y*_)_1-*x*_O_1-*v*_films have the advantages of large magnetization and non-hysteresis of magnetization at relatively small magnetic field.

## Methods

### Film growth

CoMgZnO thin films of 100 nm, together with MgO buffer layers of 10 nm, were deposited on SrTiO_3_ (001) and Al_2_O_3_ (0001) substrates by radio frequency oxygen plasma assisted molecular beam epitaxy. The background pressure of the growth chamber is better than 5 × 10^−9^ mbar. Before deposition, the SrTiO_3_ and Al_2_O_3_ substrates were thermally annealed at 800 °C for 10 minutes in growth chamber, and then the epitaxial films were deposited at 400 °C. All the studied films were grown on SrTiO_3_, except those for optical measurements, which were grown on Al_2_O_3_. Metal fluxes were provided by evaporating high purity elemental solid sources (5N cobalt, 3N8 magnesium and 6N zinc), and O flux was supplied in form of active oxygen (5N5) radicals by an RF plasma source. Composition ratio of Mg and Zn was controlled to achieve the *fcc* structure wherein high Co concentration is available. In the present Co_*x*_(Mg_*y*_Zn_1-*y*_)_1-*x*_O_1-*v*_ thin films, *x* ranges from 0.1 to 0.5, and *y* ranges from 0.55 to 0.70.

### Structure characterization

The whole growth process was monitored by reflection high energy electron diffraction (RHEED), and the crystal structure was characterized by X-ray diffraction (XRD) and high resolution transmission electron microscopy (TEM). Film thickness was estimated by *in-situ* quartz crystal monitors and the composition was checked by X-ray photoelectron spectroscopy (XPS).

### Magnetic measurements

Magnetic properties of the films were measured by superconducting quantum interference device (SQUID), and the magnetic signal from substrate was deducted. Electron holography experiments were carried out to observe the magnetic flux contours of the film surface using a JEM2100F TEM with a custom-made field-free objective lens (residual field <5 Oe).

### Energy band characterization

X-ray absorption spectrum (XAS) measurements were performed at Beamline 8.0.1 of Advanced Light Source (ALS) at Lawrence Berkeley National Laboratory (LBNL). The undulator and spherical grating monochromator supply a linearly polarized photon beam with resolving power up to 6000. The experimental energy resolution is 0.1–0.15 eV. Data were collected in both total electron yield (TEY) and total fluorescence yield (TFY) modes. All the spectra have been normalized to the beam flux measured by the upstream gold mesh. Optical transmittance spectra measurements were conducted by an UV-visible dual-beam spectrophotometer to investigate the band gap of films.

## Additional Information

**How to cite this article**: Zhu, D. *et al*. Oxygen vacancies controlled multiple magnetic phases in epitaxial single crystal Co_0.5_(Mg_0.55_Zn_0.45_)_0.5_O_1-*v*_ thin films. *Sci. Rep.* **6**, 24188; doi: 10.1038/srep24188 (2016).
